# Examination of motor skill competency in students: evidence-based physical education curriculum

**DOI:** 10.1186/s12889-017-4105-2

**Published:** 2017-02-23

**Authors:** Weiyun Chen, Austin Hammond-Bennett, Andrew Hypnar

**Affiliations:** 1 School of Kinesiology, University of Michigan, 1402 Washington Heights, Ann Arbor, MI 48109 USA; 2 Livonia Public Schools, 15125 Farmington Road, Livonia, MI 48154 USA; 3 Fairfax County Public Schools, Fairfax, VA USA

**Keywords:** Motor skill assessment, Motor skill competency, and quality physical education

## Abstract

**Background:**

Researchers found that children with a competent level of motor skill performance are more likely to be physically active. This study examined how well K-1 students demonstrated motor skill competency in relation to Physical Education Content Standard 1.

**Methods:**

Participants were K-1 grade students (*N* = 1,223-1,588; boys = 568–857; girls = 526–695; Mean age = 5.5 yrs old) who were enrolled in nine elementary schools. The K-1 students’ motor skill competency in running, weight transferring, hand dribbling, and underhand catching skills was assessed using four PE Metrics skill assessment rubrics in the intervention year 1 and year 2, respectively. Data were analyzed by means of descriptive statistics and independent sample t-tests.

**Results:**

The students in the intervention year 1 and year 2 cohorts performed at the Competent Level or higher in the four skill assessments. The prevalence of the students’ demonstration of skill competency across the four skills was high in the two intervention years. The intervention year 2 cohort scored significantly higher than the intervention year 1 cohort in the four skill assessments. The boys significantly outperformed than the girls in the two manipulative skills in the intervention year 1 and in the two manipulative skills and the weight transferring skill in the intervention year 2. No gender differences in the running skill in either year were found.

**Conclusions:**

The evidence-based CATCH PE play a critical role in developing and building K-1 students’ ability to demonstrate motor skill competency in four fundamental skills.

**Trial registration:**

ClinicalTrials.gov ID: NCT03015337, registered date: 1/09/2017, as "retrospectively registered".

## Background

Fundamental movement skills lay the foundations for children to participate in a variety of physical activities and sports [1–7]. Fundamental movement skills consist of locomotor skills (motor skills that move the body from one place to another, e.g., running, jumping), manipulative skills (motor skills that use hands, feet, or body parts to manipulate or control an object, e.g., dribbling with hands, catching, throwing), non-manipulative skills (motor skills that are performed with the body or body parts without manipulating any objects, e.g., weight transferring, balancing) [8–13]. Children’s demonstration of motor skill competency contributes to their physical and motor development. Motor skill competency is defined as “the ability to perform various fundamental movement skills (FMS) in a consistent and proficient manner”. ([14], p.2).

Empirical studies showed that motor skill proficiency was positively associated with physical activity participation and negatively linked to sedentary behaviors in children [1, 3, 6]. Children in the highest quartile of motor skill proficiency spent significantly more time participating in moderate-to-vigorous physical activity than did children in the lower levels of motor skill proficiency. Furthermore, childhood manipulative skill proficiency significantly predicted whether adolescent would participate in any moderate-to-vigorous activity and spend time in organized activity, compared to locomotor skills [7]. Also, studies showed that childhood mastery of fundamental movement skills contributed to their adolescents’ learning and mastering specialized skills needed for successful participation in sports and physical activity [1, 7]. The high-skilled children were more likely to engage in physical activities, while low-skilled children were less likely to participation in physical activities during their adolescent years [1, 4–7]. Given the fact that participation in physical activity declines as the adolescent years compared to childhood [15, 16], it is of critical importance to improving motor skill competency in both childhood and adolescents in order to maintain adherence to physical activity participation during adolescence.

The studies supported the importance of developing children’s motor skill competency during elementary school years [14, 17–22]. Children’s motor skill development is not an absolute function of physical growth. How well the motor skill is developed and mastered depends on how coherently the learner interacts with the task and the environment. In other words, children’s motor skill development occurs best when children learn and practice the skill through engaging in sequential learning tasks within structured learning environment based on children’s sequence of motor skill development [11, 12, 23]. To help students demonstrate competency in motor skills and movement patterns [24, 25], physical education teachers should provide students with a quality physical education program, which is a powerful vehicle for equipping children with competency in motor skills and movement patterns [11–13, 23–25]. Quality physical education provides students with a variety of developmentally appropriate and fun physical and fitness activities and uses effective instructional strategies to maximize students’ learning time and participation in MVPA during a PE lesson [23–31].

The Child and Adolescent Trial for Cardiovascular Health (CATCH) was a comprehensive school-based physical education and nutrition program funded by National Heart, Lung, and Blood Institute of National Institute of Health (NIH) [8, 10]. Over the course of the past three decades, the CATCH Physical Education (PE) component has been evolved into a standards-based PE curriculum. The CATCH PE aimed at improving motor skill competency, enhancing health-related fitness, and promoting positive attitudes toward participation in physical activities. In a study [26], the CATCH PE intervention took place in 96 elementary schools located in four U.S. cities (San Diego, California; New Orleans, Louisiana; Minneapolis, Minnesota; and Austin, Texas). The results showed that the students in the CATCH PE intervention schools spent 51.9% of lesson time engaging in MVPA during lessons, while those in the control schools spent 42.3% of lesson time in MVPA. The intervention schools engaged in significantly more MVPA than the control schools at *p* < .01 [26]. The scope and sequence of CATCH PE maps most of the essential content addressed by the national standards and grade-level outcomes for K-12 Physical [23–26, 32, 33]. Mapping standards-based meaningful physical education content, CATCH PE offers 500 motor skills and physical fitness activities on 20 modules that are developmentally appropriate for elementary school students [32, 34]. However, how the current CATCH PE is conducive to improving grades K-1 students’ motor skill competency remains largely unexplored.

The national physical education content standard 1 in the US stated that students should “demonstrate competency in motor skills and movement patterns” as a result of participation in a quality physical education program [24]. To date, the PE Metrics: Assessing the National Standard 1: Elementary [15] was designed to assess levels of students’ competency in motor skills and movement patterns based on the benchmarks of the national physical education content standards. Over the course of 5 years’ testing motor skill competency of 4,000 students at over 90 schools in the USA using the PE Metrics [10], it is empirically evident that the PE metrics are sound psychometric assessment rubrics used for measuring and evaluating grades K-1 students’ motor skill competency in selected locomotor skills and manipulative skills with both process- and product-oriented criteria [10]. Detailed criteria for each skill assessment rubric is shown in methods section. However, due to a lacking of performance-based assessment tools in previous studies, motor skill competency was evaluated either using product-oriented criteria with a combined product score [28] or merely process-oriented criteria with “yes” or “no” rating scale (e.g. Test of Gross Motor Developement-2) [1–3, 6].

To date, few studies used both process- and product-based assessment tools to assess whether or not young children at age of 5–6 demonstrated motor skill competency [11]. This study examined how well K-1 students demonstrated motor skill competency as a result of participating in CATCH PE. Specifically, this study aimed at examining (a) levels and proportions of K-1 students’ demonstration of competency in running, dribbling with hands, underhand catching, and weight transferring skills in the partial implementation of CATCH PE year and the implementation of CATCH PE year; (b) differences of students’ motor skill competency in the four skills between the partial implementation of CATCH PE year and the implementation of CATCH PE year; and (c) gender differences of motor skill competency in the four skills in the partial implementation of CATCH PE year and the implementation of CATCH PE year. To the best of our knowledge, this is the first study to comprehensively examine the levels of motor skill competency demonstrated by K-1 students using the selected grade-specific assessment rubrics of the PE Metrics [9].

## Methods

### Participants

In this study, during the partial implementation of CATCH PE year (intervention year 1), the number of participating K-1 students who were assessed with each of the four motor skill assessments ranged from 1,223–1,588 (boys = 654–857; girls = 536–695; Mean age = 5.5 yrs old). During the implementation of CATCH PE year (intervention year 2), the number of participating K-1 students who were assessed with each of the four motor skill assessment ranged from 1,227–1,277 (boys = 568–730; girls = 526–642; Mean age =5.6 yrs old). They were enrolled in nine elementary schools located in Mid-West of the United States. The student population was dominantly White and non-Hispanic (91.2%). In the nine elementary schools, K-1 students had a 30-min PE class and a 30-min wellness class per week taught by their certified PE teacher at his/her school. All nine PE teachers (five female and four male) were Caucasian. At the time of this study, the PE teachers’ ages varied from 33–55 years old with a range of 6–26 years of teaching experience. Each PE teacher had a spacious gymnasium with a climbing wall. The typical PE class had 18–28 students. The university institutional review board approved the project (HUM00088758) and the school district granted the permission for conducting this study. The parent/guardian of K-1 students signed the consent form for approving their child’s participation in this study.

### CATCH PE training and implementation

To examine the students’ motor skill competency in four skills, this study used the pre- and post-test research design. During the project year 1 (teacher training and preparation phase), the nine PE teachers were trained in CATCH PE curriculum during a two-day staff development workshop. To ensure quality training for all PE teachers to be able to implement CATCH PE lessons [8], four essential components including Opportunity to Learn, Meaningful Content, Appropriate Instruction, and Student and Program Assessment were used as a guiding principles for the CATCH PE trainings. In the full-day CATCH School Implementation Training Workshop, the PE teachers learned the best instructional practices echoing the four essential components of the high-quality physical education through engaging in hands-on CATCH PE lesson activities. They discussed strategic approaches to incorporating CATCH PE into their current PE programs. Two weeks later, in a full-day staff development, the nine PE teachers reviewed and discussed the CATCH PE curriculum guidebooks for K-5. Based on their shared discussion and consensus, they designed the action plan for implementing a couple of CATCH PE units for the upcoming intervention year 1. Meanwhile, a training for assessing quality teaching practices and PE Metrics assessment was held in order to help the teachers have a better understanding of what quality teaching practices look like in a PE lesson and be able to effectively conduct the PE Metrics assessments with their students. All nine physical education teachers learned critical teaching components within four essential dimensions of quality teaching and PE Metrics assessment rubrics, assessment criteria, assessment tasks, and testing protocols for selected skills.

During the partial implementation of CATCH PE year (year 1), each PE teacher began teaching CATCH PE lessons to their K-1 students (the year 1 cohort) while recording what specific CATCH PE K-1 lessons they taught using the daily Curriculum Log for K-1. According the school year calendar, each elementary schools had 72 PE lessons per school year. The PE teachers, on average, taught 37 CATCH PE lessons throughout the intervention year 1. The results indicated that CATCH PE lessons counted for 51% of PE lessons during that school year. Throughout the school year, the research team provided on-going support by conducting field observation of each PE teacher’s teaching four lessons and having immediate conversations with the teacher about his/her teaching those lessons. At the end of each CATCH PE unit, the K-1 students’ motor skill competency was assessed using a specific PE Metrics skill assessment during a regular PE class.

During the implementation of CATCH PE year (year 2), each PE teacher taught CATCH PE K-2 lessons to their K-1 students (the year 2 cohort). Similarly, based on the school year calendar, each elementary schools had 72 PE lessons. The nine PE teachers, on average, taught 55 CATCH PE lessons throughout the intervention year 2. The results showed that the CATCH PE lessons counted for 77% of the PE lessons. Besides teaching CATCH PE lessons, the nine PE teachers also taught team building lessons, CATCH Go for Health lessons, bowling lessons, and floor hockey lessons. Similarly, throughout the school year, the research team provided on-going support by observing each PE teacher’s teaching four lessons and having immediate conversations with the teacher about his/her teaching those lessons. At the end of each CATCH PE unit, the students’ motor skill competency was assessed using the same specific PE metrics skill assessment as the last year.

### Motor skill assessments

The K-1 students’ motor skill competency was assessed using four PE Metrics Assessment rubrics, including running skill, underhand catching skill, weight transferring, and hand dribbling skill assessment rubrics during the year 1 and year 2, respectively. Each rubric is designed as a skill-specific assessment tool specifically for Kindergarten and first-grade students [15]. Based on the specific feature of a skill, each skill assessment tool consists of the assessment rubrics, the assessment task, and the testing protocol. Each skill assessment rubric is comprised of essential dimensions, performance indicators, rating scales with 0–4 levels, and the number of trials for testing. The 0–4 rating scales are used to differentiate levels of motor skill performance based on defined characteristics of a performance indictor within each essential dimension on each point rating scale. Level 3 on each essential dimension indicates a competent level. A sum of the score on each essential dimension represents an overall competent level.

Regarding the running skill assessment, when a student was running within a lane over the course of 60 feet in length, his/her running performance was assessed on the two essential dimensions: Form and Consistency of Action with a 0–4 rating scale. Criteria for Competence (Level 3) on the Form are: “Runs with the essential elements of a mature pattern: a) arm/leg opposition, b) toes point forward, c) arms swing forward/backward and do not cross midline of body, and d) fee land heel to toe”. ([15], p. 41) Criteria for Competence (Level 3) on the Consistency of Action is “Runs in straight pathway without stumbling, stopping or falling down”. ([15], p. 41) One trial was allowed for this assessment. A total score of 6 indicated an overall competent level. The maximum score is 8. For the underhand catching assessment, a student used an underhand catching pattern to catch a ball tossed by his/her teacher who stood 6 feet away. His/her performance of this skill was assessed on the two essential dimensions: Form and Catching Success. Criteria for Competence (Level 3) on the Form are: “Attempts the catch with selected essential elements: a) hands reach to meet the ball. b) uses hands without trapping ball against chest. c) does not turn head away from ball”. ([15], p. 50) Criteria for Competence (Level 3) on the Catching Success is “Catches the ball successfully”. ([15], p. 50) Three trials were allowed for this assessment. A total score of 18 indicated an overall competent level. The maximum score is 24. For the weight transferring assessment, a student placed weight on their hands and transferred their feet sideways over a raised bar (3 feet long and 6 inches high) and back to a starting position. Each student’s performance was assessed on the two essential dimensions: Form and Weight Support and Control. Criteria for Competence (Level 3) on the Form are: “Transfer weight to hands, with selected essential elements: a) taking off on 2 fee simultaneously. b) landing on 2 feet simultaneously. c) hands maintaining stationary contact with the floor”. ([15], p. 56) Criteria for Competence (Level 3) on the Weight Support and Control is “Transfers weight to hands, without feet contacting the bar or without falling down”. ([15], p. 50) The Assessment consisted of 2 trials. A total score of 12 indicated overall competent level. The maximum score is 16. For the hand dribbling assessment, a student continuously dribbled the ball for 15 s with one hand within a 3-foot square taped on the floor. Each student’s performance in hand dribbling was scored on the two essential dimensions: Form and Continuous Action and Control. Criteria for Competence (Level 3) on the Form are: “Dribbles with all the selected essential elements: a) one-hand contact. b) maintains constant height of rebound. c) pushes ball (no slapping)”. ([15], p. 25) Criteria for Competency (Level 3) on the Continuous Action and Control is “Maintains a continuous dribble with feet staying within boundaries”. ([15], p. 50) One trial was allowed. A total score of 6 indicated overall competent level. The maximum score is 8. The Cronbach alpha reliability coefficients of underhand catching, running, weight transferring, and hand dribbling were 0.68, 0.81, 0.89, and .95.

### Data analysis

Descriptive statistics were used to analyze levels and prevalence of the students’ motor skill competency in the four skill assessments. To examine the mean score difference on the overall competent level between the intervention 1 cohort and the intervention year 2 cohort, an independent *t*-test was conducted for each skill assessment. Also, an independent *t*-test was utilized to examine the differences of motor skill competency in the four skills between boys and girls in the intervention year 1 cohort and the intervention year 2 cohort, respectively. An alpha level .05 was set for all tests. IBM SPSS Statistics 21 was used to conduct the statistical analyses.

## Results

As seen in Table 1, the results of this study indicated that on average, the year 1 cohort’s mastery of the running skills was on the competent level (*M* = 6.50, *SD* = 1.20). 77.5% of the students (1,231 out of 1,588) reached the competent level or above. The year 2 cohort’s mastery of the running skills, on average, were higher than the competent level (*M* = 6.71, *SD* = 2.48). 83.1% of the students (1,020 out of 1,227) demonstrated the competent Level or above. For the dribbling skill, the results showed that on average, the year 1 cohort’s mastery of dribbling skill was on the competent level (*M* = 6.13, *SD* = 1.63). 65.5% of the students (906 out of 1,384) exhibited the competent level or above. In the year 2, the students’ mastery of the skill, on average, was on the competent level (*M* = 6.38, *SD* = 1.64). 72.1% of the students (911 out of 1,263) reached the competent level or above. With respect to weight transferring skill, the results indicated that on average, the year 1 cohort’s mastery of weight transferring skill was the competent level (*M* = 12.37, *SD* = 2.72). 69% of the students (849 out of 1,223) demonstrated the competent level or above.

**Table 1:** Students’ performance in four motor skills in the intervention year 1 and year 2

	N	# of students	% of students	Score at CL	M	SD
		(≥ CL)	(≥ CL)			
Year 1						
Running	1588	1231	77.5%	6	6.50	1.20
Dribbling	1384	906	65.6%	6	6.13	1.63
Weight Transferring	1223	849	69.4%	12	12.37	2.72
Underhand Catching	1445	1050	72.2%	18	19.34	3.76
Year 2						
Running	1227	1020	83.1%	6	6.71	2.48
Dribbling	1263	911	72.1%	6	6.38	1.64
Weight Transferring	1237	1083	87.6%	12	14.00	3.75
Underhand Catching	1277	1047	81.9%	18	20.35	3.72

*CL* Competent Level

In the year 2, the results showed that on average, the students’ mastery of the skill was two point higher than the competent level (*M* = 14.00, *SD* = 3.75). 87.6% of the students (1,083 out of 1,273) demonstrated the competent level or above. Regarding the underhand catching skill, the year 1 cohort’s mastery of the skill, on average, was more than one point higher than (*M* = 19.34, *SD* = 3.76). 72.2% of the students (1,050 out of 1,445) exhibited the competent level and above. In the year 2, the students’ mastery of the skill, on average, was more than two points higher than the component level (*M* = 20.35*, SD* = 3.72). 81.9% of the students (1,047 out of 1,277) reached the competent level or above.

As presented in Table 2, the results of the *t*-test revealed a significant mean difference at the overall competent level in the four skill assessments between the two cohort groups. The year 2 cohort scored statistically and significantly higher than the year 1 cohort on the running skill (*t* = − 2.976, *df* = 2812), the hand dribbling skill (*t* = − 3.82, *df* = 2645), the weight transfer skill (*t* = − 12.345, *df* = 2458), and the underhand catching skill (*t* = − 7.045, *df* = 2720) at *p* < .01.

**Table 2:** Results of independent t-tests for the four skills between the two cohorts

Skill Tests	*t* (equal variances not assumed)	*df*	Sig. (2 tails)
Running	−2.976	2812	.000
Dribbling	−3.819	2645	.000
Weight Transferring	−12.345	2458	.000
Underhand Catching	−7.045	2720	.000

Table 3 illustrates the mean scores of overall skill competency in the four skill assessments by gender in the year 1 cohort. The results of the *t*-test yielded a significant mean difference at the overall competency between the boys and the girls in the hand dribbling (*t* = 3.46, *df* = 1382, *p* < .01), and the weight transferring skill (*t* = 2.208, *df* = 1219, *p* < .05). However, the results of the *t*-test yielded no significant gender differences in the running skill (*t* = 1.331, *df* = 1585), and the underhand catching skill (*t* = .302, *df* = 1324) at *p* > .05

**Table 3:** Results of independent t-tests for the four skills between the two cohorts by gender

Skill Tests	M	SD	t*	*df*	Sig. (2 tails)
	(Boys vs Girls)	(Boys vs Girls)			
Year 1					
Running	6.54 vs 6.46	1.20 vs 1.20	1.331	1585	.183
Dribbling	6.27 vs 5.97	1.65 vs 1.59	3.462	1382	.001
Weight Transferring	12.53 vs 12.19	2.71 vs 2.72	2.208	1219	.027
Underhand Catching	19.01 vs 18.95	3.67 vs 3.68	.302	1324	.762
Year 2					
Running	6.68 vs 6.75	1.22 vs 3.30	-.490	1225	.624
Dribbling	6.85 vs 6.29	1.50 vs 1.65	5.835	1070	.000
Weight Transferring	14.66 vs 14.21	4/93 vs 2.31	2.276	1050	.024
Underhand Catching	20.59 vs 20.12	3.68 vs 3.75	2.286	1275	.022

*t* = equal variances not assumed

Table 3 presents the mean scores of overall skill competency in the four skill assessment by boys and girls in the year 2 cohort. The results of the *t*-test indicated that there was a significant mean difference at the overall skill competency between the boys and the girls in three skill assessments. These were the hand dribbling (*t* = 5.835, *df* = 1070, *p* < .01), the weight transferring skill (*t* = 2.267, *df* = 1050, *p* < .05), and the underhand catching (*t* = 2.286, *df* = 1275, *p* < .05). In contrast, the results of the *t*-test yielded no significant gender difference in the running skill (*t* = −.49, *df* = 1225, *p* > .05).

## Discussion

The first aim of this study was to examine the levels and proportions of K-1 students demonstrate competency in motor skills in relation to the national physical education content standard 1. In the partial implementation of CATCH PE year (year 1), on average, the students demonstrated slightly higher than the competent level on the running, hand dribbling, and weight transferring skills, and performed moderately higher than the competent level on underhand catching skill. In the implementation of CATCH PE year (year 2), the students, on average, demonstrated slightly higher than the competent level on the running and the hand dribbling skills, and quite higher than the competent level on the weight transferring and the underhand catching skills. This study showed promising results, compared to the study by Zhu et al. [35] who conducted a polite study of PE Metrics assessments reliability and validity. The study reported that 264 Kindergarten students who completed the running skill test demonstrated lower than the competent level (*M* = 2.64, *SD* = .76). 562 Kindergarten students who completed the hand dribbling skill test demonstrated nearly one level lower than the competent level (*M* = 2.08, *SD* = 1.13). 150 Kindergarten students who completed the weight transferring skill test demonstrated between the competent level and the incompetent level (*M* = 2.58, *SD* = .79). 202 Kindergarten students who completed the underhand catching skill test exhibited slightly lower than competent level (*M* = 2.9, *SD* = .87) [33].

Further, this study shows that as the implementation of CATCH PE lessons increased from the year 1 to year 2, the percentage of students who reached the competent level or above in the four skills also increased. For example, in the year 1 (partial implementation of CATCH PE year), the proportions of students who demonstrated the competent level or above in the four motor skills were high, ranging from 65.5% for the hand dribbling, 69% for the weight transfer, 72.7% for the catching, to 77.5% for the running. However, more encouraging results were found in the year 2 (implementation of CATCH PE year). The percentage of students who demonstrated the competent level or above in the four motor skills was ranged from high to very high, 72.1% for the hand dribbling, 81.9% for the catching, 83.1% for the running, to 87.6% for the weight transfer. The results of this study were much higher than the results in previous studies [2, 36]. For example, Okely and Booth [2] examined young children’s mastery/near mastery of four locomotor skills and two object control skills assessed using the process-oriented assessment checklists with two-point “yes” or “no” rating scale in three years. The results indicated that in all three year groups (mean age was 6.2, 7.2, 8.2 in year 1, 2, and 3), the students’ master of a skill was lower than 35% n [2]. In contrast, this study indicated that the majority of the K-1 students adequately achieved the National Physical Education Standard 1 in the four fundamental movement skills. In contrast, only a small portion of the students in this study needed to improve their competency in these motor skills. Corroborating with previous studies [26–31, 36–42], this study suggests that a well-designed PE curriculum plays one of critical roles in improving motor skill competency among young children.

In line with previous studies which showed that boys performed better with object control skills [1–5, 40], this study indicated that the year 1 boys significantly outperformed than the year 1 girls in the hand dribbling skill. Also, the year 2 boys performed significantly better in the hand dribbling and catching skills than the year 2 girls. Studies [1, 18] reported that object control skills were more significant contributors for girls to engage in organized physical activities compared to boys. Especially, girls with highly proficient sport skills continued their participation in that sport, in contrast, girls with poor sport skills were less likely to participation in a sport during their adolescence. This study suggests that it is of critical importance to improving girls’ basic specialized manipulative skills used in playing sports.

The results of this study were consistent with previous studies which found no gender difference in the running skill [41, 42]. However, inconsistent with previous studies that indicated girls were better with locomotor skills [1–5, 18, 40], this study showed that the boys in the two year cohorts significantly outperformed than the girls in weight transferring skills. Regardless of gender differences, both the boys and the girls in the two year cohorts demonstrated higher than the competent level in the two locomotor skills (running and weight transfer) and two manipulative skills (hand dribbling and catching), except for the girls in the year 1 cohort demonstrated close to the competent level in the hand dribbling. Previous studies showed that physical activity participation was more strongly related to locomotor skills than to object-control skills among young children [3–5, 10, 18]. The children who demonstrated higher levels of locomotor skill competency participated in more moderate to vigorous physical activities and decreased their sedentary behaviors, compared to children with lower levels of locomotor skill competency [3–5, 10, 18]. This study indicated that the boys and the girls possessed well-developed locomotor skills. Their locomotor skill competency would be helpful for them to successfully play various types of age-appropriate games and enjoy physical activities during a physical education lesson and in recess.

Research also showed that children with object control skill proficiency are more likely to play organized and non-organized sports and to participate in specific physical activities during their adolescent years [1, 7, 10, 40]. Given the declining trend of physical activity participation among adolescents [15, 16, 43], it is critical to develop children’s motor skill competency in manipulative skills. The boys and girls in this study were well equipped to demonstrate competency in the two manipulative skills, and especially the catching skills. Developing mastery of catching skill is instrumental for boys and girls in order to play a variety of sports during their upper elementary years and secondary years. Development of competency in manipulative skills is a stepping stone to help learn physical education content with greater success and enjoyment in adolescence [7, 9, 10]. This study suggests that PE teachers need to provide more learning opportunities for children to learn and practice a variety of manipulative skills such as hand dribbling, dribbling with feet, kicking, overarm throwing, underhand throwing, volleying with body parts, striking skills with rackets and bat. Mastery of these manipulative skills will successfully lead to students’ learning basic specialized skills used in playing a variety of sports [11, 12].

It is noted that the limitation of this study is related to the research design. Due to the fact that this study did not use an experimental research design, this study did not have a control group. Therefore, this study was limited to examining the levels and proportion of K-1 students’ demonstration of competency in running, weight transferring, underhand catching, and hand dribbling skills in the two CATCH PE cohort groups. Accordingly, this study focused on reporting the results of students’ demonstration of motor skill competency in the four skills in the year 1 cohort (partial implementation CATCH PE year) and in the year 2 cohort (implementation of CATCH PE year). Further, to have a better understanding of the extent to which CATCH PE lessons would contribute to students’ demonstration of motor skill competency, using an experimental research design (i.e., randomized controlled trial, or the quasi experimental research design) in future studies should be warranted. Given the enabling role of motor skills in daily PA behaviors, a future study may examine relationships between CATCH PE and daily PA behaviors mediated by motor skill competency.

## Conclusions

The evidence-based CATCH PE plays a critical role in developing and building K-1 students’ ability to demonstrate motor skill competency, the NASPE content standard 1. The K-1 students in the intervention year 1 and year 2 demonstrated the competent level in all four motor skill assessments. The intervention year 2 cohort performed significantly better than the intervention year 1 cohort in all four motor skill assessments. The boys performed significantly better than the girls in the two manipulative skills in the intervention year 1 as well as better than the girls in the two manipulative skills and the weight transferring skill in the intervention year 2. No gender differences in the running skill assessments in both years were found.

## Abbreviations

CATCH PE: CATCH physical education curricular
CATCH: the child and adolescent trial for cardiovascular health
PE: physical education
